# Three-dimensional partitioning of resources by congeneric forest predators with recent sympatry

**DOI:** 10.1038/s41598-019-42426-0

**Published:** 2019-04-15

**Authors:** Julianna M. A. Jenkins, Damon B. Lesmeister, J. David Wiens, Jonathan T. Kane, Van R. Kane, Jake Verschuyl

**Affiliations:** 1 USDA Forest Service, Pacific Northwest Research Station, Corvallis Forestry Sciences Lab, Corvallis, OR 97331 USA; 2 Oregon State University, Department of Fisheries and Wildlife, Corvallis, OR 97331 USA; 3 US Geological Survey, Forest and Rangeland Ecosystem Science Center, Corvallis, OR 97331 USA; 4 University of Washington, School of Environmental and Forest Sciences, Seattle, WA 98105 USA; 5 National Council for Air and Stream Improvement, Anacortes, WA 98221 USA

## Abstract

Coexistence of ecologically similar species can be maintained by partitioning along one or more niche axes. Three-dimensional structural complexity is central to facilitating resource partitioning between many forest species, but is underrepresented in field-based studies. We examined resource selection by sympatric northern spotted owls (*Strix occidentalis caurina*), a threatened species under the US Endangered Species Act, and nonnative barred owls (*S*. *varia*) in western Oregon, USA to explore the relative importance of canopy heterogeneity, vertical complexity of forest, and abiotic features to resource selection and identify potential differences that may facilitate long-term coexistence. We predicted that within home range selection of understory densities, measured with airborne lidar, would differ between species based on proportional differences in arboreal and terrestrial prey taken by each owl species. We used discrete choice models and telemetry data from 41 spotted owls and 38 barred owls monitored during 2007–2009 and 2012–2015. Our results suggested that while both species used tall canopy areas more often than low canopy areas, spotted owls were more commonly found in areas with lower tree cover, more developed understory, and steeper slopes. This is the first evidence of fine-scale partitioning based on structural forest properties by northern spotted owls and barred owls.

## Introduction

Classic niche theory expresses that coexistence of ecologically similar species can be maintained over time through resource partitioning, whereby competitors vary how, when, or where they attain resources necessary for their survival^1–3^. Even in seemingly homogenous environments, subtle environmental gradients or heterogeneity enable species to differentiate resource acquisition. In forest communities, 3-dimensional structural complexity has long been recognized as important for facilitating animal assemblages^4^ and resource partitioning. For example, 5 *Setophaga* warblers in the boreal forest of North America partitioned foraging regions within coniferous trees based on height above ground and distance to tree bole^5^. Elsewhere, 11 species of *Anolis* lizard in Puerto Rico partitioned forest habitats via height and size of perch substrates^6^. Most classic examples of coexistence through resource partitioning developed along evolutionary time scales. The potential for competition is higher when species have coexisted for shorter periods^3^.

The frequency of novel competitive interactions have increased as highly mobile generalist species expand their geographic ranges via introductions or changes in climate, phenology, or landscape composition^7^. An influx of novel invasive competitors may incite rapid ecological change for native species, depending upon their degree of ecological overlap and resource specialization^3^. Species that have evolved without a high degree of interspecific competition are affected most strongly by novel competitive processes, as exemplified by interactions between native Eurasian red squirrels (*Sciurus vulgaris*) and the introduced eastern grey squirrel (*S*. *carolinensis*) in Great Britain^8^. However, it is rare that 2 species completely overlap; thus landscape heterogeneity can lead to maintenance of a weaker competitor on the landscape if some conditions favor the weaker competitor or are of sufficiently low quality to reduce competitive pressure by the dominant species^3,9^. Identifying factors that may promote partitioning of resources, or indicate qualities of potential refugia for native species, are key aspects of effective conservation strategies for threatened and endangered species in changing environments.

Over the last 50 years, the barred owl (*Strix varia*) has expanded its geographic range to completely overlap that of the threatened, congeneric, northern spotted owl (*S*. *occidentalis caurina*, hereafter spotted owl). Spotted owls are obligates of mature and late-successional forest for multiple life-history requirements^10^. Because of contemporary and historical loss of old forest, and competition with encroaching barred owls, spotted owl populations have continued to decline despite broad scale conservation efforts^11^. Sympatric predators typically differ in their size, habitat use, or prey selection. Although spotted owls evolved within a rich native owl community, they did not have a direct competitor in their size or foraging class prior to barred owl invasion^12^. Both spotted and barred owls demonstrate selection for structurally diverse forest conditions^13–15^, and both species are territorial with high site fidelity and seasonally dynamic home ranges^13^. Barred owls are considered as the superior competitor based on their more aggressive behavior, higher survival and fecundity, and broader use of forest types and prey items^11–13^.

Studies of resource competition between spotted owls and barred owls have found some support for interspecific partitioning of abiotic landscape features such as slope, topographic position, and elevation^13–15^. However, aside from subtle differences in the proportional use of old forest (used more heavily by spotted owls) and hardwood riparian forest (used more by barred owls), prior analyses have found few differences in the use of vegetative resources between the species^13^. Partitioning of habitat strata has been recorded for other congeneric owl species, and is likely a function of foraging behavior and food-niche partitioning. For example, tawny owls (*S*. *aluco*) and Ural owls (*S*. *uralensis*) in Poland differ in their use of canopy densities and edge areas^16^, and the elegant scops-owl (*Otus elegans*) and Japanese scops-owl (*O*. *semitorques*) appeared to partition resources along vertical space in terms of the type of prey most frequently taken (arboreal vs. terrestrial)^17^. While partitioning of vertical space is a well-documented mechanism for co-existence in forest birds, it has yet to be examined for barred owls and spotted owls.

Past comparisons of spotted owl and barred owl resource selection used broad categorizations of vegetative communities while ignoring much of the heterogeneity of vertical structures within forest stands. Until recently, studies utilizing fine-scale metrics of vegetation structure were rare for species inhabiting large spatial areas, due to the difficulty and effort required when measuring vertical variation^18^. Metrics of forest structure from light detection and ranging (lidar) data are a promising tool to study fine-scale forest partitioning because they can sample landform and vegetative structures accurately across large areas at fine resolutions^18,19^. Lidar-derived metrics have already successfully been used to evaluate suitability of forests for spotted owl nesting^20,21^ and habitat for owl prey species^22^.

Diet studies comparing the proportions of prey taken by sympatric spotted and barred owls support interspecific differences in primary foraging strata^9,23^. Spotted owls captured greater proportions of arboreal prey (e.g. tree squirrels, tree voles), while barred owls captured greater proportions of terrestrial prey (e.g. shrews, mice, and insects). Given that the distribution and density of arboreal, scansorial, and terrestrial prey species differ within forests based upon vegetative structure and complexity^24,25^, we predicted that selection of forest structural features within home ranges would also differ between barred owls and spotted owls. We further predicted that differences in use of forest structure would be reduced in the nonbreeding (winter) season when terrestrial–based prey (e.g. aquatic and insect sources) consumed by barred owls, but not by spotted owls, may be more limited. We used nighttime locations derived from telemetry data and metrics of 3-dimensional forest structure derived from airborne lidar data to test our predictions. Specifically, we compared the relative importance of structural features of forest canopy, sub-canopy, and abiotic features for ‘breeding’ versus ‘nonbreeding’ resource selection by barred owls and spotted owls to infer whether the two species partitioned the use of these conditions in a manner that may facilitate sustained coexistence.

## Results

**Figure 1:**
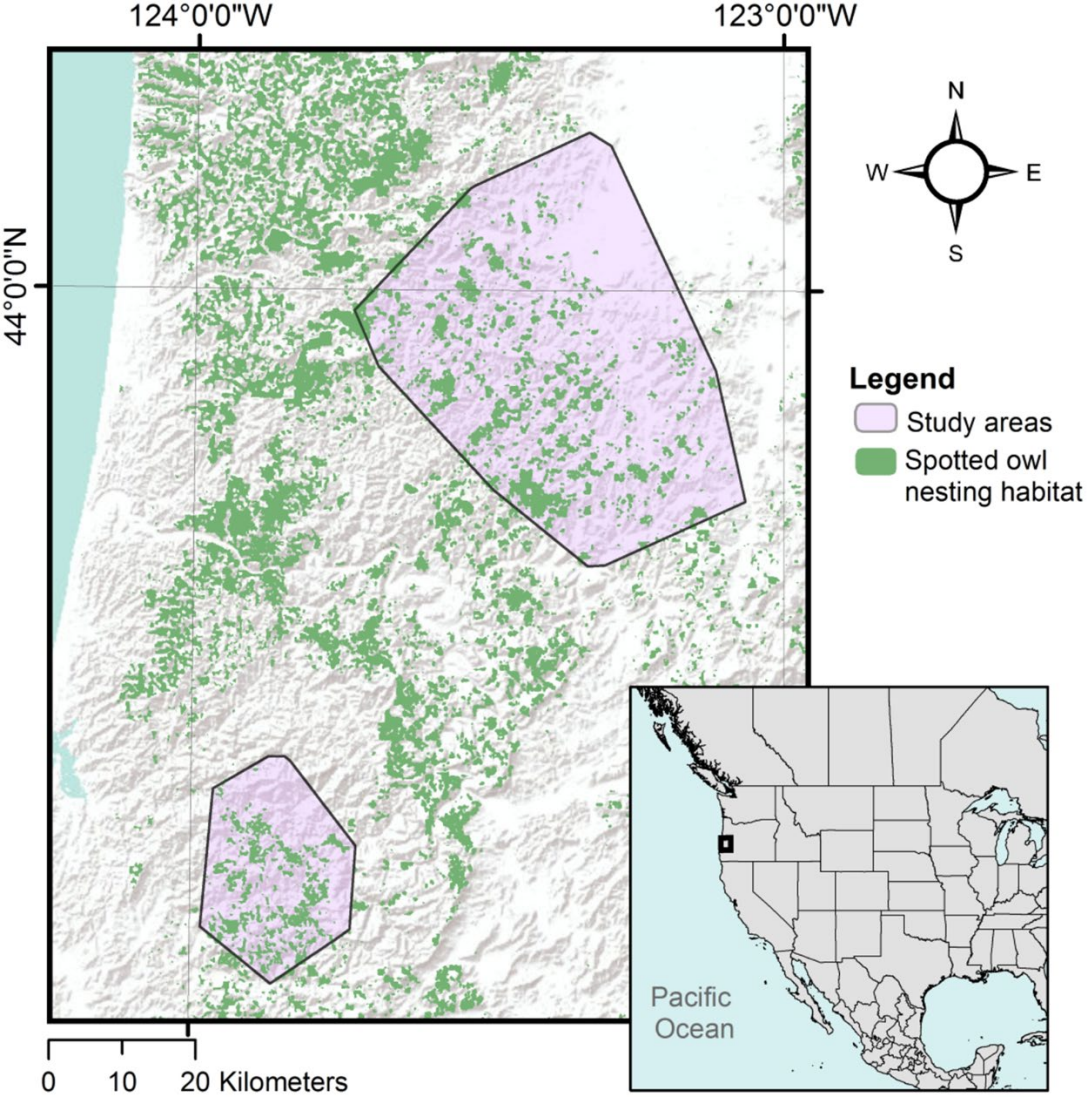
Study area map. Telemetry of northern spotted owls and barred owls occurred on two study areas in western Oregon, USA in 2007–2009 (north area) and 2012–2014 (south area). The nesting/roosting habitat for northern spotted owls generated by Glenn *et al*. (2017) is shown in green.

We used telemetry data from 41 adult territorial spotted owls (19 females and 22 males) and 38 adult territorial barred owls (19 females, 19 males) acquired across 7 breeding seasons and 5 nonbreeding seasons from 2007–2013 in western Oregon, USA (Fig. 1). Discrete choice models utilized 3,975 choice sets (19,875 random locations) from spotted owls and 4,060 choice sets (20,300 random locations) from barred owls. Seventy-eight percent of owl relocations occurred at night. There were 1,144 areas (9.89 ± 12.28 ha; range: 1.08–55.53 ha), representing 6% of the total study area, which were not included in the analysis due to timber harvests occurring between telemetry and lidar acquisition.

**Figure 2:**
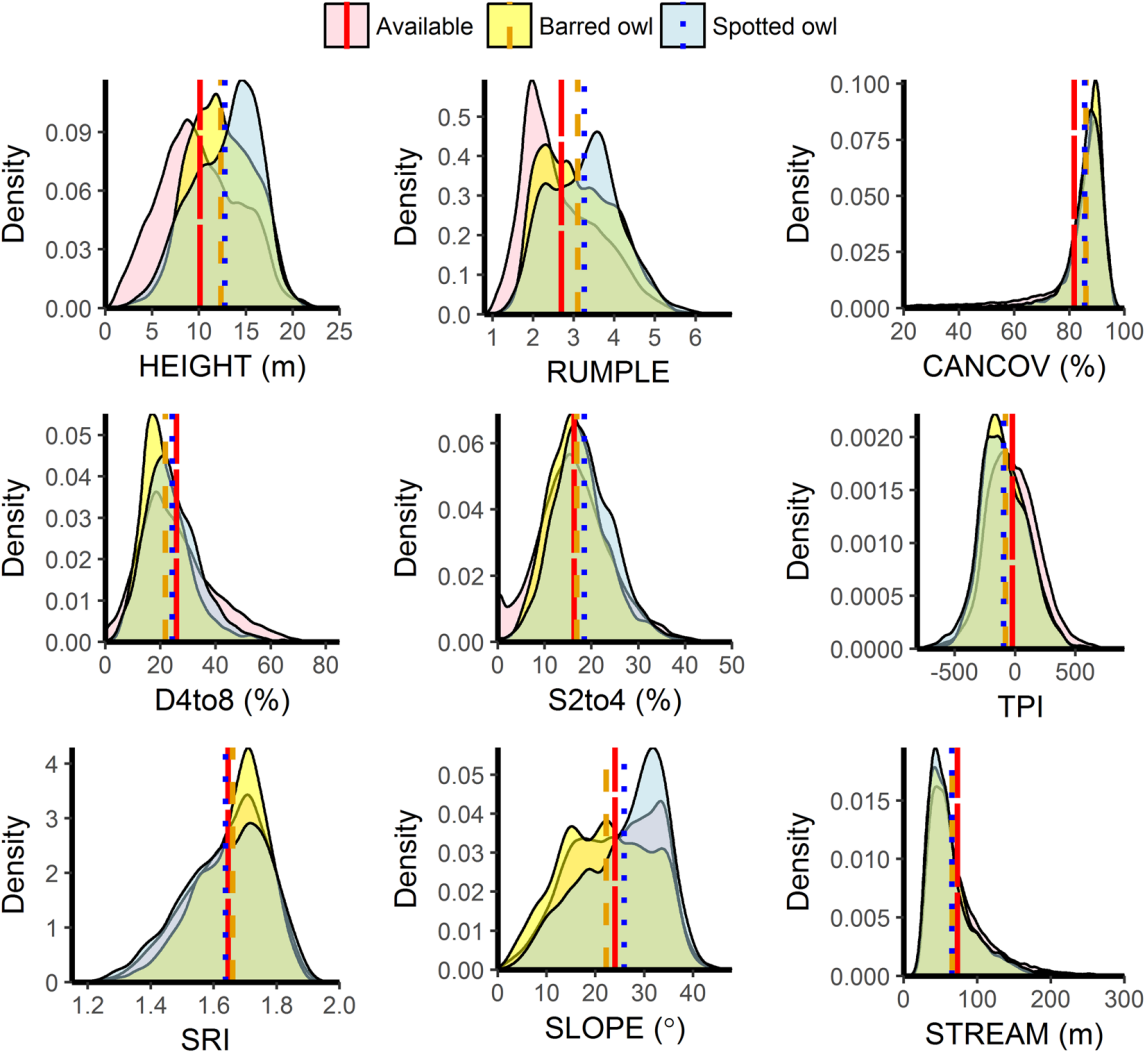
Distributions of environmental metrics at random (n = 40175), barred owl (n = 4060), and spotted owl (n = 3975) locations in western Oregon, USA. Vertical lines represent sample means. The three sample means were statistically different (*p* < 0.01) in Welch 2 sample t-test comparisons for all metrics except STREAM, where barred owl and spotted owl use differed from random (*p* < 0.01) but not between species (*p* = 0.46). Abbreviations: HEIGHT, dominant canopy height; RUMPLE, rumple index; CANCOV, canopy cover >2 m; D4to8, cover from 4–8 meters; S2to4, sub-canopy cover from 2–4 meters; STREAM, distance to nearest permanent stream; SRI, solar radiation index; TPI, topographic position index.

The mean value of environmental conditions at both spotted owl and barred owl locations were greater than mean values calculated at available locations for sub-canopy strata cover 2–4 m (S2to4), canopy cover (CANCOV), RUMPLE and HEIGHT and less than the available sample for topographic position index (TPI), distance to stream (STREAM), and strata cover 4–8 m (D4to8; Fig. 2). Solar radiation index (SRI) sample means were similar for all samples and SLOPE sample means were greater than the available sample mean for spotted owls and less than the available sample for barred owls (Fig. 2).

The full discrete choice model containing HEIGHT (HEIGHT + CANCOV + HEIGHT × CANCOV + D4to8 + S2to4 + HEIGHT × D4to8 + SRI + TPI + TPI^2^ + SLOPE + STREAM) received the most support in model comparisons (Table 1). Some parameters did not fully converge among three independent chains in 10-fold cross-validation model training, but parameter estimates were consistent when models were fitted repeatedly. The top model scored 35% in ten-fold cross-validation (2x greater than the model of random selection, 17%) with better performance for spotted owl locations (41% of breeding and 37% nonbreeding) than barred owl locations (31% of breeding and nonbreeding). We considered this performance highly satisfactory given our conservative available choice sets that incorporated locations from within the use location’s home range and thus likely encompassed lower variation within the choice set compared to the broader study areas.

**Table 1:** Ranked candidate models of resource selection by northern spotted owls and barred owls in Coast Range of western Oregon, USA.

Rank	Model covariates	Explanation	K	ΔWAIC
1	HEIGHT + CANCOV + HEIGHT × CANCOV + D4to8 + S2to4 + HEIGHT × D4to8 + SRI + TPI + TPI^2^ + SLOPE + STREAM	full model with canopy structure HEIGHT	11	0
2	RUMPLE + CANCOV + RUMPLE × CANCOV + D4to8 + S2to4 + SRI + TPI + TPI^2^ + SLOPE + STREAM	full model with canopy structure RUMPLE	10	215.84
3	HEIGHT + CANCOV + HEIGHT × CANCOV + SRI + TPI + TPI^2^ + SLOPE + STREAM	canopy structure HEIGHT and abiotic	8	236.05
4	RUMPLE + CANCOV + RUMPLE × CANCOV + SRI + TPI + TPI^2^ + SLOPE + STREAM	canopy structure RUMPLE and abiotic	8	535.10
5	HEIGHT + CANCOV + HEIGHT × CANCOV + D4to8 + S2to4 + HEIGHT × D4to8	canopy structure HEIGHT and vertical variation	6	1745.70
6	D4to8 + S2to4 + SRI + TPI + TPI^2^ + SLOPE + STREAM	vertical variation and abiotic	7	1970.64
7	RUMPLE + CANCOV + RUMPLE × CANCOV + D4to8 + S2to4	canopy structure RUMPLE and vertical variation	6	2137.13
8	HEIGHT + CANCOV + HEIGHT × CANCOV	canopy structure HEIGHT	3	2451.31
9	RUMPLE + CANCOV + RUMPLE × CANCOV	canopy structure RUMPLE	3	2882.56
10	SRI + TPI + TPI^2^ + SLOPE + STREAM	abiotic features	5	3102.12
11	D4to8 + S2to4	vertical variation	2	4069.30
12	Null	selection is random	0	5839.80

Rumple index and dominant canopy height had a Spearman’s rank coefficient ≥0.7 and were not included in the same model. The top model had a Watanabe-Akaike information criterion (WAIC) of 22953.8. Abbreviations: K, number of variables in model; HEIGHT, dominant canopy height; RUMPLE, rumple index; CANCOV, canopy cover >2 m; D4to8, cover from 4–8 meters; S2to4, sub-canopy cover from 2–4 meters; STREAM, distance to nearest permanent stream; SRI, solar radiation index; TPI, topographic position index.

**Figure 3:**
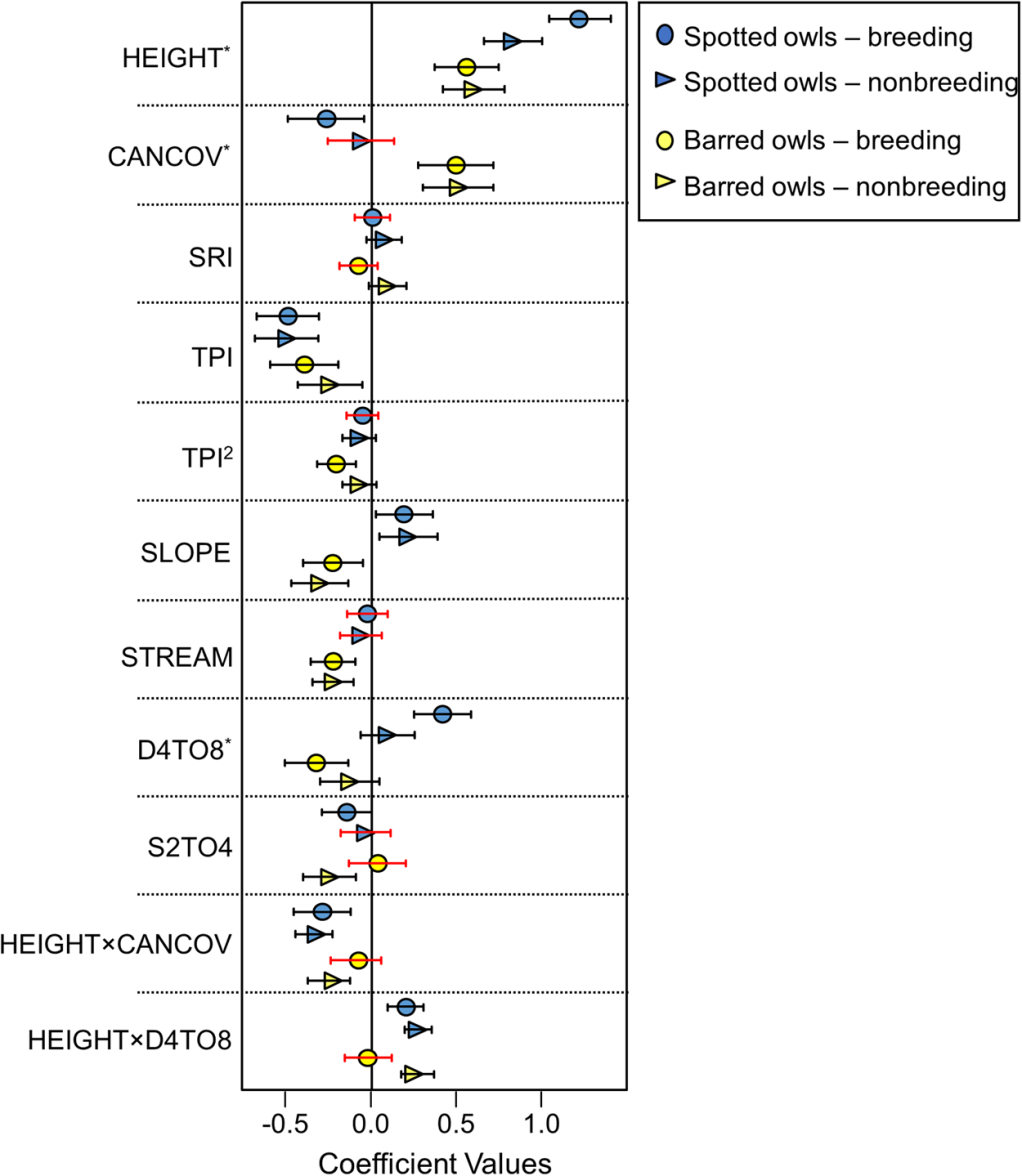
Parameter coefficients from top owl resource selection model. Mean species parameter coefficients and 95% credible intervals (error bars) for spotted owls and barred owls in breeding and nonbreeding seasons in western Oregon, USA. Red error bars indicate cases where <90% of the posterior was the same sign as the mean, suggesting low confidence in effect. Covariates with asterisk are involved in an interaction.

Breeding and nonbreeding coefficient posterior distributions for spotted owls overlapped by ≥40% for all covariates except for HEIGHT (3% overlap) and D4to8 (2.5% overlap, Fig. 3). Coefficient posterior distributions estimated for barred owls overlapped by ≥30% for all supported covariates (Fig. 3). HEIGHT was positively associated with relative probability of selection (P) for both species (Fig. 3) and was the most influential covariate for both species when variables were ranked by their relative numeric change in P (Supplementary Fig. S1). The interaction terms HEIGHT × D4to8 and HEIGHT × CANCOV were supported for breeding and nonbreeding resource selection for spotted owls, but only supported for nonbreeding resource selection by barred owls (Fig. 3, Supplementary Table S1). When covariates were held constant at their mean values, the direction of effect coefficients (positive or negative) on P was conserved between seasons for both owl species (Supplementary Fig. S2).

**Figure 4:**
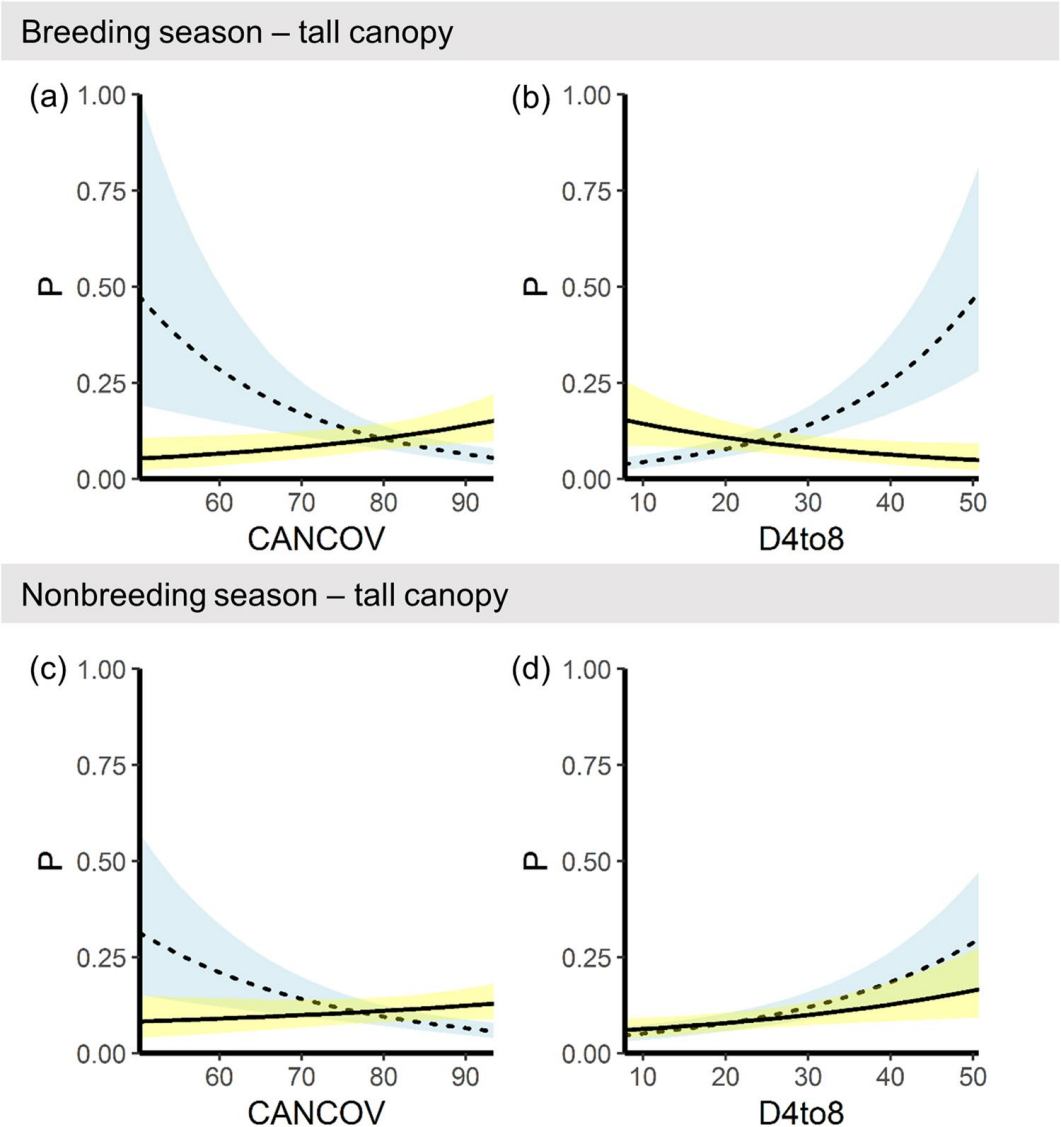
Species-specific effects of canopy cover and understory cover on resource selection in taller forest stands. Relative probability of selection (P) of tall canopy areas by spotted owls (dashed line) and barred owls (solid line) in the breeding and nonbreeding seasons in western Oregon, USA. Predictions were generated across the 5^th^ to 95^th^ percentile range of observed canopy cover (CANCOV) and relative density of lidar point cloud from 4–8 m (D4to8) while holding dominant canopy height at the 95^th^ percentile value (17.3 m) and all other covariates at their mean.

The effect of SLOPE had a nearly equal, but opposite effect on P for each species in both the breeding and nonbreeding seasons (Fig. 3, Supplementary Table S1). At the 5th percentile SLOPE (9°), barred owl relative probability of use was 2.2x greater than spotted owl P (P = 0.41 and P = 0.18, respectively) and at the 95^th^ percentile SLOPE (36°) spotted owl P was 2.3x greater than barred owl P (P = 0.36 and P = 0.16, respectively) when other covariates were held constant (Supplementary Fig. S2). CANCOV was more influential to barred owl than spotted owl selection (Fig. 3, Supplementary Fig. S2). When dominant canopy was tall (e.g. 95^th^ percentile: 17.3 m), CANCOV was negatively associated with selection by spotted owls, but positively associated with selection by barred owls (Fig. 4).

In the breeding season, D4to8 was the third ranked variable in relative importance for selection for both species (Supplementary Fig. S1). When covariates were held constant at their mean values, breeding season spotted owls were more likely to select areas with high D4to8 (40–50%) whereas breeding season barred owls were more likely to select areas with low D4to8 (10–20%; Fig. 4). However, in areas with tall canopy (e.g. 95^th^ percentile: 17.3 m) during the nonbreeding season, the relative probability of use for both barred and spotted owls was positively associated with D4to8 (Fig. 4).

## Discussion

Here we provide the first evidence for resource partitioning between spotted owls and barred owls based upon 3-dimensional structural properties of forest. We found that both species used areas with tall dominant canopies most often, but use within these conditions by each species differed in relation to finer-scale canopy cover and understory density. Spotted owls selected for areas with less canopy cover in areas where the dominant canopy was tall (>10 m), whereas barred owls were more associated with higher canopy cover regardless of tree height. Spotted owls were positively associated with cover of the understory from 4–8 m in tall forest (canopy height >10 m) in both seasons. Conversely, barred owls selected areas with lower understory cover from 4–8 m in the breeding season and higher understory cover from 4–8 m in the nonbreeding season.

We suggest two alternative, but not mutually exclusive hypotheses of the underlying drivers for these divergent selection patterns: niche partitioning and niche compression. First, differences in foraging behaviors between the two owl species may help explain differential use of canopy cover and understory vegetation densities in areas with tall trees. The combination of tall trees, higher rumple values, and lower canopy cover are effective measures of complex old-growth stands^19^. Multi-layered canopy forests, common in old-forest conditions, tend to have greater densities of arboreal mammalian species that are primary prey of spotted owls, such as the red tree vole (*Arborimus longicaudus*)^22^ and northern flying squirrel (*Glaucomys sabrinus*)^25^. Additionally, the shift observed in our study by barred owls from a negative association with vegetation cover between 4–8 m during the breeding season (opposite spotted owls selection), to a positive association in the nonbreeding season (matching spotted owl selection) aligns with the increase in interspecific overlap in dietary composition previously documented in our northern study area from 45% in the breeding season to 68% during the nonbreeding season^13^. These findings and ours suggests that seasonal changes in prey availability (and vegetation characteristics) are relevant to resource partitioning between these recently sympatric avian predators.

Second, the selection for forests with greater density of understory vegetation may reflect a behavioral response of spotted owls to antagonistic interactions and potential predation by barred owls, thereby reducing direct competitive interactions with the slightly larger and more aggressive *Strix* species. Selection for direct overhead cover as a mechanism to reduce competition and predation risk has been reported for owl prey and other small forest predators^26,27^. The majority of our observations came from nighttime telemetry locations when owls were primarily foraging, but likely also engaging in other behaviors such as roosting. While we cannot make direct comparisons to pre-invasion vertical strata selection by these species, as comparable data are not available, historical records indicate that prior to invasion, spotted owls roosted in low strata locations during the breeding season and higher canopy areas during the nonbreeding season to aid thermoregulation^28^. Spotted owls may be experiencing barred owl-driven niche compression in space use by spending more time in areas of dense understory between foraging bouts to reduce agonistic interactions with barred owls.

Terrain conditions were identified as influential for habitat use by barred owls in the eastern Cascade Range of Washington^14^ and from prior analyses in our study area^9^. We found that slope differentiated fine-scale resource use between species, where spotted owls used steeper slopes compared to barred owls. Differential use of slope conditions may reflect the spotted owl’s lower wing-loading which may allow them to more effectively maneuver and hunt in steep slope areas^29^. Both spotted and barred owls had a negative relationship to TPI, suggesting both species selected drainage bottoms or depressions where available, however distance to permanent stream was negatively related to barred owl selection, suggesting barred owls were more likely to select locations within larger drainages. A study tracking spotted owls between 1995 and 1998, in the north coast range of Oregon, found evidence that spotted owls foraged within riparian forests^30^. We found no evidence for within home range selection based on distance to stream, at the level of choice sets. The lack of spotted owl selection for distance to stream observed in our study may signal that niche compression away from riparian stream beds is occurring. Areas of steep slope and favorable forest structural conditions may act as fine-scale refugia for spotted owls in the presence of competing populations of barred owls.

When a novel competitor enters an area, some changes in distribution and population size of the initial occupant are expected. From a conservation perspective, it is critical to identify resource conditions which may promote regional co-occurrence between natives and intruders. The differences in fine-scale forest structure associations observed in this study aligned with previous observations of dietary differences, and could indicate partitioning or reflect niche compression effects where spotted owls have been displaced from some foraging habitats as a result of competitive interactions with barred owls. Forest and owl prey communities vary across the range of the spotted owl^31,32^, so the differential use of fine-scale forest structural metrics observed in our study should be considered as a basis for further hypothesis testing, perhaps within an adaptive management framework. Differences in selection within tall canopy forests suggest that vegetation manipulation of understory canopies might provide a mechanism to mitigate effects of competitive interactions with barred owls on populations of northern spotted owls, however further study is needed to link structural metrics from lidar to forest community composition and management history.

## Methods

### Owl Telemetry

Telemetry data on adult territorial spotted owls and barred owls were collected in two sampling areas in the Coast Range of western Oregon (Fig. 1). The habitat model generated by Glenn *et al*. (2017)^32^ identifies approximately 22% of each study area as suitable for nesting and roosting by spotted owls, and barred owls in the region were present at densities 3–8x higher than spotted owls^33^. Both the north and south study areas had extensive road systems with high ridges, and consisted of a patchwork of lands managed by the US Bureau of Land Management (40% and 52%, respectively), the Oregon Department of Forestry (2% and 1%, respectively), and private land holders including large timber companies (58% and 47%, respectively).

Territorial adults of both owl species were captured in spatially associated nesting areas using standardized field protocols and equipped with backpack-style radio transmitters. Radio-marked owls were monitored using directional handheld antennas and portable receivers, and relocated 1–5 times per week at night (0.5 hr after sunset to 0.5 hr before sunrise; see Wiens *et al*. (2014) and Irwin *et al*. (2018) for additional details)^13,34^. We also relocated owls at their daytime roosts at least once per week in the northern study area^13^. All animal methods were approved by the Animal Care and Use Committee of Oregon State University and performed in accordance with the relevant guidelines and regulations from the US Fish and Wildlife Service and Oregon Department of Fish and Wildlife.

### Environmental metrics

Data from five airborne lidar acquisitions collected from 2009 to 2015 were used in the study (Supplementary Table S2). We used the USDA Forest Service’s FUSION lidar processing software (http://forsys.cfr.washington.edu/fusion/fusionlatest.html), version 3.60^35^ to process the lidar data to produce metrics at 30 m resolution describing both the forest canopy structure and topography (Table 2). Details on the algorithms used to calculate the canopy structure and topography metrics are available in the FUSION manual^35^. We selected 8 FUSION metrics (5 biotic, 3 abiotic) which we considered easily interpreted and which have either been associated with increasing structural complexity of forest in our region^19,36^ or related to habitat features proposed to influence owl or owl prey distribution on the landscape.

**Table 2:** Environmental metrics used as covariates in discrete choice models of resource selection for northern spotted owls and barred owls in western Oregon, USA.

Structure	Variable	Description
Canopy variation	CANCOV	Canopy cover- percent of lidar point cloud returns >2 m above ground
	HEIGHT	Dominant canopy height- height above ground (m) at which 95% of lidar point cloud returns fall below
	RUMPLE	Rumple index- a measure of vertical and horizontal canopy height complexity where higher values are correlated with greater forest structural complexity
Vertical variation	D4to8	Density (cover) from 4–8 m [# lidar returns 4–8 m / # lidar returns <8 m]
	S2to4	Sub-canopy density (cover) from 2–4 m [# lidar returns 2–4 m where HEIGHT >4 m / # returns <4 m]
Abiotic features	SRI	Solar radiation index- describes solar radiation theoretically striking an arbitrarily orienting surface around noon on the equinox
	TPI	Topographic position index- compares the elevation of each cell in a surface to the mean elevation of a 2,000 m neighborhood around that cell
	SLOPE	Mean hill slope (degrees)
	STREAM	Distance to nearest permanent stream (m) based on data from national hydrology database (https://nhd.usgs.gov)

Dominant canopy height (HEIGHT), canopy cover above 2 m (CANCOV), and rumple index (RUMPLE) have been associated with increasing structural complexity of old growth forest structure^19^. To represent HEIGHT for each 30 m pixel, we calculated the height at which 95% of returns >2 m height above ground lie below. CANCOV was calculated as the count of returns greater than 2 m above ground divided by the total count of all returns^35^. RUMPLE is a measure of the rugosity of the outer canopy surface and ground. RUMPLE was calculated as the ratio of the area of the 1 m resolution canopy surface model, computed using the maximum return height with each 1 m grid cell and smoothed with a 3 × 3 low pass filter, to the planar projection of the area of the underlying digital terrain model for each 30 m grid cell^35^. We utilized canopy cover in 2 sub-overstory strata as proxy measures of structural density in the mid- and under-story^29^. We selected two strata to represent canopy cover from taller shrubs, shorter trees, and possibly the lower branches of overstory trees: cover in the 4–8 m strata (D4to8) and cover in the 2–4 m strata where HEIGHT > 4 m (S2to4). Both cover estimates were calculated as the count of returns in that stratum (2–4 m or 4–8 m) divided by the sum of the count of returns in that stratum and all lower strata^35^. We chose 2–4 m and 4–8 m as strata bounds because they were below the mean HEIGHT and were not correlated with other vegetative measures of interest.

The FUSION software package calculated slope, a solar radiation index (SRI), and a topographic position index (TPI) based on Jenness (2006) algorithm^35,37^. TPI was calculated using windows of 200 m, 500 m, 1,000 m, and 2,000 m. In addition to the lidar generated metrics, we measured distance to nearest permanent stream using data from the US national hydrology database (https://nhd.usgs.gov/NHDPlus_HR.html). We used ArcGIS 10.3.1 (ESRI Redlands, CA, USA) to extract the mean values for each environmental metric within 150 m of points, which was the mean error of telemetry locations^13^.

### Resource selection analysis

We modelled within home range resource selection for the breeding season (March 1–August 31) and the nonbreeding season (September 1–February 28) separately to account for differences in owl behavior associated with nesting and feeding of young^10^ and differences in prey between phenological seasons^10,31^. We used multinomial logit discrete choice models in a hierarchical Bayesian framework to model resource selection within home ranges in a use-availability design^38,39^. Discrete choice models estimate the probability that a single unit with certain characteristics will be selected during one choice ocassion^40^. Discrete choice models are appropriate for dynamic systems where availability changes between individuals or over time because ‘used’ resources are only compared to resources available within a ‘choice-set’^40^. Choice-sets in our analysis consisted of an owl’s triangulated (used) location and 5 paired random ‘available’ points taken from within the individual bird’s seasonal home range (described below). Since choice sets are customized to each ‘choice’ occasion, variation caused by changes in factors that are constant across all choices within a choice set (such as sex, phenological season, or year) are removed, similar to “blocking” nuisance variation in analysis of variance^40^.

To delineate available resources, we generated 95^th^ percentile fixed-kernel home ranges using program GME 0.7.4.0 with a likelihood cross-validation smoothing parameter^41^. When both members of a pair were monitored, we generated the pair’s breeding season home range jointly. We only generated home ranges for owls with >27 relocations per season because of instability of kernel estimates with small sample sizes^42^. Owl home ranges often included private lands managed for timber production (i.e., thinning or clear-cut harvests). Because some lidar was not collected concurrently with owl data (Supplementary Table S2), we used digital maps of forest change^43^ (http://landtrendr.forestry.oregonstate.edu) to identify areas within 150 m of land harvested between the date of telemetry locations and lidar acquisition. Any use points falling within the censored harvest areas were censored from analyses. We constrained paired random points to occur within a seasonal home range area, buffered by the average daily movement rate (520 m for spotted owls and 350 m for barred owls). All points within choice sets (1 use & 5 random) were at least 300 m apart and were not within censored areas.

We used an information-theoretic approach and the Watanabe-Akaike information criterion (WAIC) to evaluate and rank a candidate set of competing models^44^. The candidate model set included a null model of uniform likelihood and *a priori* models representing hypotheses on the relative importance of canopy structure, vertical variation, and abiotic features (Table 1). We assessed all pairwise correlations and did not permit covariate combinations with a Spearman’s rank coefficient of ≥0.7. Prior to building models, we ranked univariate models of TPI generated at 200 m, 500 m, 1,000 m, and 2,000 m; TPI at 2,000 m was the highest ranked scale and was used in all models incorporating abiotic covariates. We tested whether TPI and SRI were better considered as quadratic or linear effects by comparing the mean prediction rate from a 10-fold cross validation^45^. We included an interaction between HEIGHT × D4to8 in all resource selection models where these covariates co-occurred. We did this because at canopies >8 m, D4to8 represented sub-canopy vegetation cover, while at canopy heights below 8 m, D4to8 was representative of canopy density. We included the interaction HEIGHT × CANCOV and RUMPLE × CANCOV *post hoc* after initial analyses showed that the effect sign for canopy cover (CANCOV) and D4to8 changed when HEIGHT was present.

For all models, posterior distributions for each parameter were estimated using Markov chain Monte Carlo methods implemented in JAGS 4.3.1 using the jagsUI^46^ and rjags^47^ packages in program R 3.5.0^48^. We accounted for repeated observations of individuals within a season by assuming that individual-level coefficients arose from normal species-level distributions^38,39^. We selected vague priors for all model parameters and assumed normal *N* (0, 0.01) prior distributions on all regression coefficients. We examined three chains for each model and ran chains until the Brooks-Gelman-Rubin convergence diagnostic suggested adequate convergence ($\hat{R}$ ≤ 1.1). After convergence, we sampled 2,000 draws from the joint posterior of each chain.

We standardized variables prior to analysis such that the mean selection ratio (exp[β]) for variables not involved in an interaction or quadratic effect represented the effect of one standard deviation change in the relative probability of selection (P). We considered coefficients of model covariates to be strongly supported when 90% of the posterior was the same sign as the mean (*f* ≥ 0.9). To assess the relative importance of covariates in the best supported model, we estimated relative response curves for each variable while holding other covariates at their mean values. We evaluated the predictive ability of the top model using a modified k-fold cross-validation design^45^. We divided the data into 10 random testing subsets, each containing 10% of all choice sets, then successively removed each testing subset and refit the model using the remaining data. The average percentage of testing data where selection within choice sets (6 locations within home range) was correctly assigned represented the predictive score of the model; predictive scores higher than the random model (17%) were considered successful.

## Supplementary information

Supplementary Tables and Figures

## Data Availability

The full datasets examined during the current study are available from the corresponding author on reasonable request.
