# Supplementary material for: Three-dimensional partitioning of resources by congeneric forest predators with recent sympatry
Source: Sci Rep. 2019 Apr 15;9:6036. doi: 10.1038/s41598-019-42426-0 (PMC6465400; doi:10.1038/s41598-019-42426-0)
Supplement: Supplementary file 1 — Supplementary Tables and Figures [file 41598_2019_42426_MOESM1_ESM.docx]

**Supplementary Information**

**Three-dimensional partitioning of resources by congeneric forest predators with recent sympatry.**

Julianna M. A. Jenkins, Damon B. Lesmeister, J. David Wiens, Jonathan T. Kane, Van R. Kane, and Jake Verschuyl

**Supplementary Table S1.** **Posterior top model coefficient means (β), standard deviation (sd) and the proportion of the posterior with the same sign as the mean (*f*) from models of northern spotted owl and barred owl resource selection for the breeding season and nonbreeding season in Western Oregon.** Higher values of *f* (approaching 1) represent increasing confidence in the direction of the covariate effect. Abbreviations: HEIGHT, dominant canopy height; CANCOV, canopy cover >2m; D4to8, cover from 4–8 meters; S2to4, sub-canopy cover from 2–4 meters; STREAM, distance to nearest permanent stream; SRI, solar radiation index; TPI, topographic position index

|  | Spotted owl | | | | | | |  | Barred owl | | | | | | |
| --- | --- | --- | --- | --- | --- | --- | --- | --- | --- | --- | --- | --- | --- | --- | --- |
|  | breeding | | |  | nonbreeding | | |  | breeding | | |  | nonbreeding | | |
|  | β | sd | *f* |  | β | sd | *f* |  | β | sd | *f* |  | β | sd | *f* |
| HEIGHT | 1.22 | 0.09 | 1.00 |  | 0.83 | 0.09 | 1.00 |  | 0.56 | 0.10 | 1.00 |  | 0.60 | 0.09 | 1.00 |
| CANCOV | -0.26 | 0.11 | 0.99 |  | -0.05 | 0.10 | 0.70 |  | 0.50 | 0.11 | 1.00 |  | 0.52 | 0.10 | 1.00 |
| SRI | 0.01 | 0.05 | 0.59 |  | 0.08 | 0.05 | 0.93 |  | -0.07 | 0.06 | 0.89 |  | 0.10 | 0.06 | 0.96 |
| TPI | -0.48 | 0.09 | 1.00 |  | -0.49 | 0.09 | 1.00 |  | -0.39 | 0.10 | 1.00 |  | -0.24 | 0.10 | 0.99 |
| TPI^2^ | -0.05 | 0.05 | 0.83 |  | -0.06 | 0.05 | 0.90 |  | -0.20 | 0.06 | 1.00 |  | -0.06 | 0.05 | 0.90 |
| SLOPE | 0.20 | 0.09 | 0.99 |  | 0.22 | 0.09 | 0.99 |  | -0.22 | 0.09 | 0.99 |  | -0.30 | 0.09 | 1.00 |
| STREAM | -0.02 | 0.06 | 0.60 |  | -0.06 | 0.06 | 0.82 |  | -0.22 | 0.07 | 1.00 |  | -0.22 | 0.06 | 1.00 |
| D4to8 | 0.42 | 0.08 | 1.00 |  | 0.10 | 0.08 | 0.90 |  | -0.32 | 0.10 | 1.00 |  | -0.12 | 0.09 | 0.91 |
| S2to4 | -0.14 | 0.07 | 0.97 |  | -0.03 | 0.07 | 0.66 |  | 0.04 | 0.08 | 0.70 |  | -0.24 | 0.08 | 1.00 |
| HEIGHT×CANCOV | -0.28 | 0.09 | 1.00 |  | -0.32 | 0.06 | 1.00 |  | -0.07 | 0.08 | 0.77 |  | -0.22 | 0.06 | 1.00 |
| HEIGHT×D4to8 | 0.21 | 0.05 | 1.00 |  | 0.28 | 0.04 | 1.00 |  | -0.02 | 0.08 | 0.63 |  | 0.26 | 0.05 | 1.00 |

**Supplementary Table S2.** Lidar acquisitions used to generate environmental variables for models of northern spotted owl and barred owl resource selection for the breeding season and nonbreeding season in Western Oregon.

| Acquisition | Willamette Valley | South Coast | Rogue River | Lane County | Upper Umpqua |
| --- | --- | --- | --- | --- | --- |
| Area (ha) | 804,033 | 660,600 | 551,074 | 504,197 | 572,254 |
| Year data acquired | 2009 | 2009 | 2012 | 2013 | 2015 |
| Vendor | Watershed Sciences | Watershed Sciences | Watershed Sciences | Watershed Sciences | Quantum Spatial |
| Instrument family | Not reported | Not reported | Leica ALS | Leica ALS | Optech Orion & Leica ALS |
| mean pulses/m^2^ | 8.14 | 8.1 | 10.36 | 10.15 | 12.96 |

**Figure S1.** **Relative influence of variables explaining relative probability of selection (P) by spotted owls and barred owls in western Oregon, USA.** Bars indicate the maximum numeric change in functional response (ΔP) predicted from the 5^th^ to 95^th^ percentile value when other variables were held constant. Abbreviations: HEIGHT, dominant canopy height; CANCOV, canopy cover >2m; D4to8, cover from 4–8 meters; S2to4, sub-canopy cover from 2–4 meters; STREAM, distance to nearest permanent stream; SRI, solar radiation index; TPI, topographic position index.

**
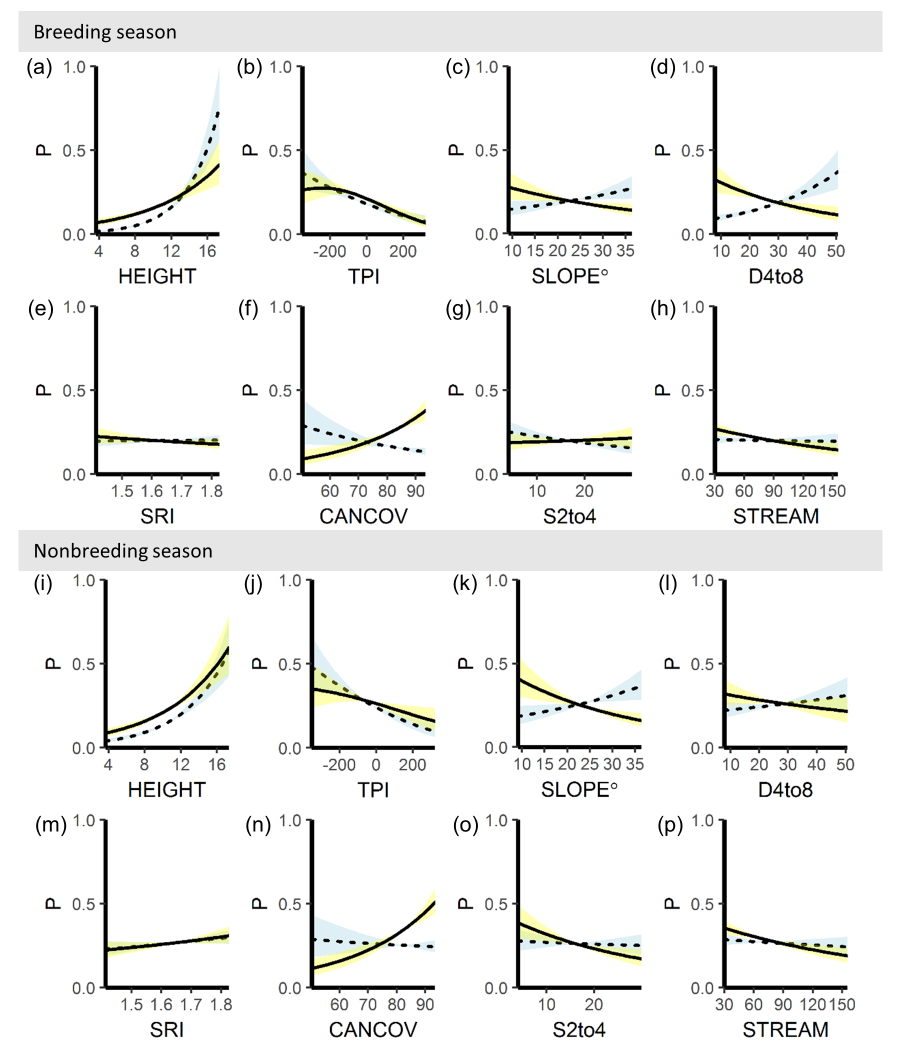
**

**Figure S2. Relative probability of selection (P) of spotted owls (dotted line) and barred owls (solid line) in western Oregon, USA with 95% credible intervals.** Predictions generated for the 5^th^ to 95^th^ percentile of observed values with all other covariates held constant at their mean value. Abbreviations: HEIGHT, dominant canopy height; CANCOV, canopy cover >2m; D4to8, cover from 4–8 meters; S2to4, sub-canopy cover from 2–4 meters; STREAM, distance to nearest permanent stream; SRI, solar radiation index; TPI, topographic position index.


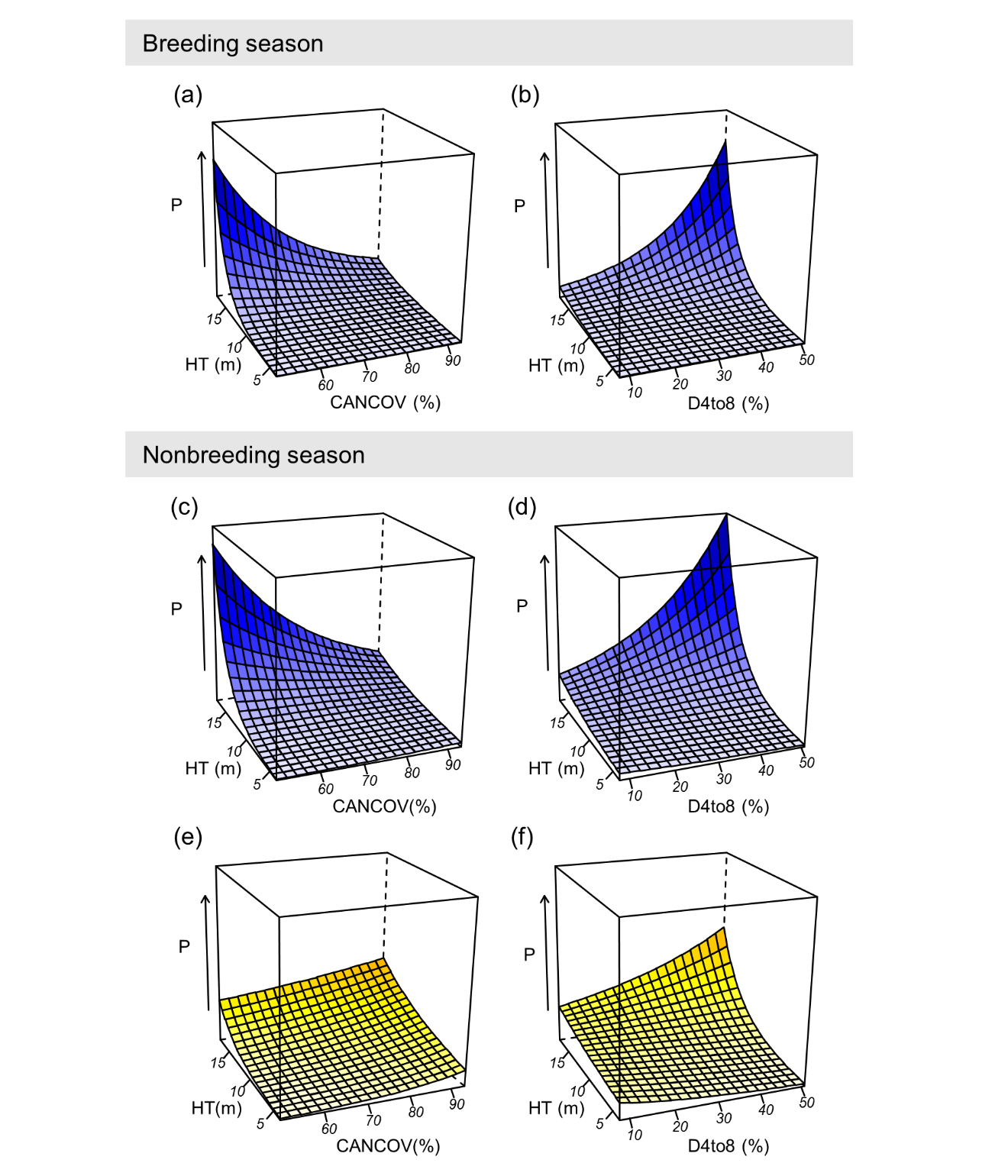


**Figure S3. Mean relative probability of selection (P) surface plots**. Predictions were generated for supported interactions of dominant canopy height (HEIGHT; HT) and density from 4–8m (D4to8) and between HT and canopy cover (CANCOV) for spotted owls (a–d) and barred owls (e–f) in western Oregon, USA. Predictions were generated across the 95^th^ percentile range of sampled conditions while holding all other covariates at their mean.
